# Supplementary material for: Accelerated nanopore basecalling with SLOW5 data format
Source: Bioinformatics. 2023 May 30;39(6):btad352. doi: 10.1093/bioinformatics/btad352 (PMC10261880; doi:10.1093/bioinformatics/btad352)
Supplement: btad352_Supplementary_Data [file btad352_supplementary_data.pdf]

SUPPLEMENTARY MATERIALS
Accelerated nanopore basecalling with SLOW5 data format

Hiruna Samarakoon[1,2,3] *, James M. Ferguson[1,2] *, Hasindu Gamaarachchi[3,1,2] #, Ira W. Deveson[1,2,4] #

[1] Genomics Pillar, Garvan Institute of Medical Research, Sydney, NSW, Australia.
[2] Centre for Population Genomics, Garvan Institute of Medical Research and Murdoch Children's Research Institute, Australia.
[3] School of Computer Science and Engineering, University of New South Wales, Sydney, NSW, Australia.
[4] Faculty of Medicine, University of New South Wales, Sydney, NSW, Australia.
* Contributed equally
# Joint-senior authors; correspondence: i.deveson@garvan.org.au & hasindu@garvan.org.au

SUPPLEMENTARY TABLES

Supplementary Table 1. Datasets used for benchmarking experiments.

Dataset description	Pore type	Reads	N files (BLOW5)	Reads per file (BLOW5)	Total file size (BLOW5)	N files (FAST5)	Reads per file (FAST5)	Total file size (FAST5)	Total seq. (Gbases)	Total signal samples (Gsamples)	Median raw signal length per read (*n* signal samples)
PromethION human genome sample (NA12878, ~30X)	R9.4.1	9,083,052	1	9,083,052	0.74TB	2272	4000	1.3 TB	93.4	1080.9	87,368
Random subset of dataset **1**	R9.4.1	500,000	1	500,000	37GB	125	4000	71 GB	5.1	56.7	80,305

Supplementary Table 2. Computer specifications.

System	Description	CPU (No. of cores/threads)	GPU type	RAM (GB)	File system	Disk system	OS
dist-sys	National Computer Infrastructure (NCI) GPU node	2 x Intel Xeon Platinum 8268 (48/96)	4 x Tesla V100-32 GB	384	Lustre	7200 4TB disks in 120 NetApp disk arrays	CentOS 8.3.2011
para-sys	Academic HPC with parallel file system	2 x Intel Xeon Silver 4114 (20/40)	4 x Tesla V100-16 GB	384	PanFS	ASH-100 12TB disks in RAID6+ configuration	CentOS 7.9.2009
cloud-sys	AWS p3.8xlarge EC2 instance	1 x Intel Xeon CPU E5-2686 v4 (16/32)	4 x Tesla V100-16 GB	240	AWS S3	-	Ubuntu 20.04.4 LTS
prom-sys	PromethION P48 compute tower (PRO-PRCV100)	2x Intel Xeon Platinum 8180 (112)	4 x Quadro GV100 - 32 GB	384	EXT4	8x8TB SSD drives with RAID0 configuration	Ubuntu 20.04.4 LTS
